# Climate Change Threats to Medicinal Plants: Progress in Impact Assessments and Implications for Pharmaceutical Sustainability

**DOI:** 10.3390/plants15132009

**Published:** 2026-06-29

**Authors:** Yixian Cheng, Zilong Zhang

**Affiliations:** School of Chinese Materia Medica, Beijing University of Chinese Medicine, Northeast Corner, Intersection of South Yangguang Street and East Baiyang Road, Fangshan District, Beijing 102488, China; chengyx539@163.com

**Keywords:** medicinal plants, climate change, impact mechanisms, adaptive management, pharmaceutical sustainability

## Abstract

With the intensification of global climate change and the increasing frequency of extreme weather events, medicinal plants are facing unprecedented challenges to their survival environments. Understanding the impacts of ecological threats on medicinal plants is crucial for formulating conservation strategies and ensuring the sustainable utilization of Traditional Chinese Medicine (TCM) resources. This study employed a scoping review methodology to systematically search databases including CNKI, Wanfang Data, and PubMed, incorporating both the Chinese and English literature. A conceptual map was constructed to analyze the response mechanisms, distribution changes, and conservation status of medicinal plants under ecological threats. The review synthesizes evidence from 65 articles retrieved from both the Chinese and international literature. Our mapping reveals that (1) ecological threats are extensively documented, with habitat loss and climate change being the primary drivers; (2) the responses of medicinal plants are mainly manifested as population decline, range shifts, and alterations in secondary metabolites; (3) current conservation efforts focus heavily on ex situ protection, while research on climate change adaptation management remains insufficient. This study systematically outlines the current research landscape regarding medicinal plants under ecological threats, revealing the characteristics and gaps in existing evidence. Future research should strengthen interdisciplinary collaboration, focusing on adaptive evolution and ecological restoration technologies to address the escalating environmental challenges.

## 1. Introduction

Medicinal plants—as a specialized group dependent on specific climatic habitats—exhibit close correlations between their survival, physiological metabolism, and accumulation of active compounds with climatic conditions. Global average surface temperature has risen by 1.1 °C above pre-industrial levels and could exceed the 1.5 °C threshold between 2030 and 2050 if emissions remain uncontrolled [1,2]. Extreme climate events such as heatwaves, drought, and abnormal precipitation will increase in frequency and intensity, continuously disrupting terrestrial ecosystems and threatening medicinal plant diversity.

Approximately 80% of the global population relies on medicinal plants for healthcare, and China alone has documented over 13,000 species [3]. Climate change is imposing severe challenges: rising temperatures degrade habitats of high-altitude species [4]; combined elevated CO_2_ and drought stress alter secondary metabolic pathways, causing 20–50% fluctuations in active ingredient content [5]; and shifting climatic zones shrink suitable habitats for authentic herbs like *Panax notoginseng* and *Rhodiola rosea*. Additional factors such as nitrogen deposition and ozone stress further disrupt growth and resource regeneration by affecting soil nutrients and photosynthetic efficiency [6].

Although current research has identified core patterns such as high temperatures shortening growth cycles and drought reducing yields, significant gaps remain: mechanisms underlying interactions of multiple climate factors are not yet fully elucidated; systematic understanding of long-term trends in genetic and functional diversity is lacking; and no unified framework has been established for conservation under different climate scenarios.

**Figure 1:**
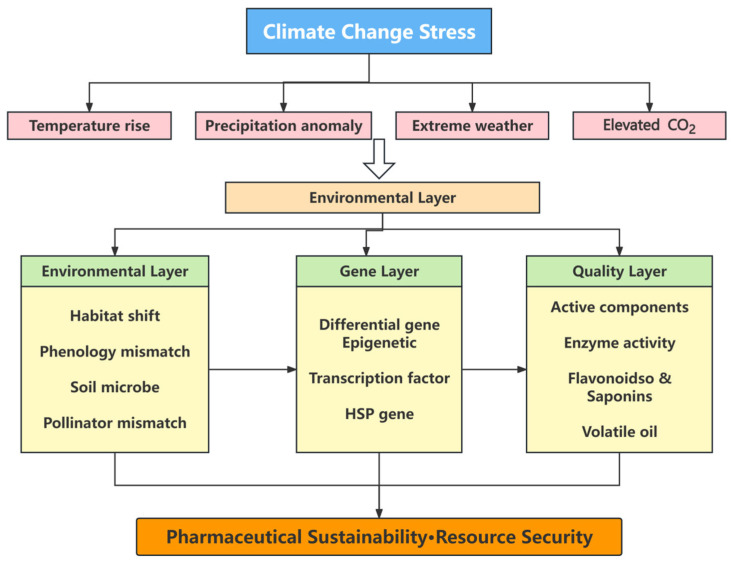
Conceptual illustration of the “Environment–Gene–Quality” cascade in medicinal plants under climate change stress. The diagram shows how climate stressors (temperature rise, precipitation anomaly, extreme weather, elevated CO_2_) sequentially or interactively affect three interconnected levels. Arrows indicate causal pathways: (1) Environmental level—changes in habitat suitability, phenology/pollinator matching, and soil microbial communities; (2) Gene level—activation of stress-responsive transcription factors (e.g., MYB, WRKY), differential gene expression (e.g., *P5CS*, *PAL*, HSP), and epigenetic modifications (DNA methylation, miRNA); (3) Quality level—alterations in bioactive compound accumulation (flavonoids, saponins, volatile oils) and enzyme activities (*PAL*, *C4H*, *SOD*). These cascading effects ultimately determine pharmaceutical sustainability and resource security. The framework is testable by integrating multi-omics data with environmental parameters. Abbreviations: HSP, heat shock protein; *PAL*, phenylalanine ammonia-lyase; *C4H*, cinnamate 4-hydroxylase; *SOD*, superoxide dismutase.

This review has four objectives: (1) to synthesize multidimensional impacts (distribution, physiology, quality, ecological interactions); (2) to elucidate physiological, molecular, and ecological mechanisms; (3) to evaluate current methodologies and their limitations; and (4) to propose an integrated “environment–gene–quality” framework and actionable adaptation strategies. This review distinguishes itself through three contributions: (i) IPCC scenario-driven multidimensional analysis of impacts from individual to ecosystem levels; (ii) proposal of a testable “environment–gene–quality” framework (as shown in Figure 1); and (iii) identification of imbalanced global research landscapes through comparative regional and species case studies. A recent review further emphasizes that environmental challenges interact with medicinal plant quality in complex, nonlinear ways, highlighting the need for systematic integration of ecological and phytochemical research [7]. Although this review takes a global perspective, China-specific climate features (e.g., accelerated warming on the Qinghai–Tibet Plateau, increased monsoon variability) are discussed in the case studies and inform the proposed adaptation strategies.

### Results

Selection of Sources of Evidence: A total of 182 records were retrieved from Web of Science, PubMed, Scopus, and CNKI (restricted to 1994–2026). After screening, 65 articles were included. Duplicates were not removed (consistent with the scoping review aim of mapping broad evidence patterns).

Characteristics of Sources of Evidence: The 65 included articles were published between 1994 and 2026. Most studies originated from Asia (particularly China). Studied species included *Panax ginseng*, *Scutellaria baicalensis*, *Gastrodia elata*, and *Rheum palmatum*, among others. The included sources comprised original research, reviews, and IPCC reports.

Critical Appraisal of Individual Sources of Evidence: No formal critical appraisal was performed, consistent with the scoping review objective of mapping the breadth of evidence rather than assessing bias.

Results of Individual Sources of Evidence and Synthesis of Results: Section 1, Section 2, Section 3 and Section 4 provide a narrative synthesis organized around four themes: (1) multidimensional impacts, (2) underlying mechanisms, (3) research methods, and (4) case studies.

## 2. Multidimensional Impacts of Climate Change on Medicinal Plants

### 2.1. Impacts on Distribution Patterns and Genetic Diversity

Global warming is reshaping the geographic distribution patterns of medicinal plants at unprecedented speed and intensity. The most pronounced manifestations include suitable habitat reduction, spatial shifts, and fragmentation. These changes reduce wild population sizes, exposing remnant populations to heightened risks of genetic drift and inbreeding depression, thereby diminishing adaptive potential and evolutionary resilience. During migration, reduced effective population size and increased geographical isolation further restrict gene flow, potentially causing allele loss and genetic diversity decline, which weakens species’ capacity to respond to future environmental changes.

According to the IPCC Sixth Assessment Report (AR6), global average temperatures have risen by approximately 1.1 °C since pre-industrial levels [1]. This change has directly caused significant retreat or displacement of the current suitable habitats for about 30% of medicinal plants, with particularly pronounced effects in high-altitude, high-latitude, and ecologically fragile zones [8]. For instance, under the SSP245 climate scenario, the optimal habitat area for *Cordyceps sinensis* in Qinghai Province is projected to decrease by 18.3% by 2050, with its habitat shifting to areas above 4200 m in the Three Rivers Source Region [9]. Similarly, the core suitable habitat for Tianshan snow lotus has shifted from the mid-mountain zone on the northern slope of the Tianshan Mountains to the high-altitude regions of the Altai Mountains at higher latitudes due to rising temperatures, while the area of unsuitable habitat has expanded by 22.6% [10]. This spatial restructuring is not solely driven by rising temperatures but is closely linked to altered precipitation patterns, frequent extreme drought events, and disrupted freeze–thaw cycles. For instance, the habitat center of six *Scutellaria* medicinal plants shifted approximately 150 km northeast due to reduced precipitation, potentially rendering their original traditional production areas ecologically unsuitable [11]. More alarmingly, habitat shifts often lag behind climate variability. Compounded by human disturbances, many species struggle to keep pace through natural dispersal, trapping them in a “climate trap.” Such drastic disruptions to distribution patterns not only threaten species survival but also undermine the ecological sustainability of traditional medicinal resources.

In summary, the dynamic restructuring of suitable habitats driven by climate change has become the foremost ecological challenge facing the sustainable utilization of medicinal plant resources. It urgently requires proactive responses through zoning adjustments and ex situ conservation strategies. Mechanistically, these distributional shifts are driven not only by rising temperatures but also by compound factors such as altered precipitation patterns, extreme drought events, and disrupted freeze–thaw cycles, which collectively determine habitat suitability and gene flow limitations. Across species, migration rates vary considerably (from <10 to >50 km per decade), and temperature is the dominant driver at high altitudes while precipitation matters more in arid zones, underscoring the need for region-specific conservation planning.

### 2.2. Impacts on Growth, Development, and Phenological Rhythms

The impact of climate change on medicinal plants is not a linear effect of a single environmental factor. Instead, it involves the synergistic interaction of multiple factors—such as coupled hydrotemperature dynamics, nutrient changes, and temperature fluctuations—forming a multidimensional physiological and ecological stress network. This synergistic mechanism primarily manifests in three aspects: growth metabolism, physiological adaptation, and phenological reproduction.

The synergistic changes in water and heat conditions constitute the core environmental factors affecting medicinal plant growth. Coupled stress from drought and high temperatures inflicts cumulative damage by disrupting cellular structures and inhibiting enzyme activity. Under moderate drought stress, *Gastrodia elata* exhibited a 19% decrease in superoxide dismutase activity in its rhizomes and a 27% increase in malondialdehyde content [12]. High temperatures further exacerbated membrane lipid peroxidation, reducing the activity of phenylalanine ammonia-lyase—a key enzyme in gastrodin synthesis—by over 32% [13]. Under combined elevated CO_2_ and reduced precipitation, *Houttuynia cordata* exhibited a 12.4% greater decline in net photosynthetic rate compared to single precipitation stress [14]. Additionally, its underground biomass allocation increased by 15% to mitigate growth pressure from water deficit. These altered physiological responses directly reduce the habitat suitability index of medicinal plants in thermohygric imbalance zones. For example, in areas experiencing prolonged drought coupled with high temperatures, the habitat carrying capacity for *Gastrodia elata* decreased by over 40%.

As an essential nutrient for plant growth, nitrogen deposition changes significantly modulate medicinal plants’ response patterns to elevated CO_2_ concentrations. In the Sanjiang Plain wetlands, *Artemisia argyi* demonstrated enhanced photosynthetic adaptation to high CO_2_ environments when nitrogen application increased by 50 kg·hm^−2^ [15]. Chlorophyll content rose by 11% compared to the no-nitrogen-addition group, and the decline in net photosynthetic rate decreased from 23% to 8%. Under high-nitrogen, low-water conditions in the Mu Us Desert, *Artemisia argyi* communities exhibited imbalanced leaf nitrogen-to-phosphorus ratios, blunting their physiological response to elevated CO_2_. The increase in community net primary productivity was 17% lower than in the water-sufficient group [16]. Nitrogen supplementation also alters nutrient accumulation in medicinal plants. For example, under high-nitrogen conditions, flavonoid synthesis in chrysanthemums from authentic production areas was suppressed, but elevated CO_2_ concentrations partially mitigated this inhibition, restoring total flavonoid content by 9% [17].

Abnormal temperature fluctuations disrupt the phenological rhythms of medicinal plants, affecting their reproductive processes and population continuity. In the Beijing-Tianjin–Hebei region, a 1 °C increase in annual mean temperature advances the flowering period of 13 medicinal plants, including peony and chrysanthemum, by 3–5 days, while delaying flowering in 11 plants, such as salvia and polygonatum. This phenological divergence reduces pollination efficiency by 15–20% [18]. Peony flower bud differentiation strictly requires accumulated low temperatures. When the duration of temperatures between 0 and 5.5 °C falls below 42 days, flower bud abortion rates increase by 35%. Rising temperatures shorten the effective low-temperature period in natural environments, further exacerbating declines in peony reproductive success [3]. In persistently elevated temperatures, ginseng exhibits significantly upregulated expression of the heat shock protein gene *PgHSP01* during flower bud differentiation. However, this cannot fully counteract high temperatures’ suppression of pollen viability, resulting in a 28% reduction in fruit set rate [19].

In summary, climate change exerts systematic synergistic effects on the growth metabolism, physiological adaptation, and phenological reproduction of medicinal plants through composite factors such as water–heat coupling, nitrogen regulation, and temperature fluctuations. A consistent pattern is that coupled water–heat stress exacerbates damage beyond additive effects, yet the magnitude differs markedly among species; phenological shifts also show directional inconsistency, with warming advancing flowering in some species but delaying it in others.

### 2.3. Effects on Active Components and Quality

Climate change triggers multi-tiered responses in medicinal plants through soil–microbial systems, molecular regulatory networks, and community ecosystems, collectively shaping adaptive capacity in changing environments.

Extreme water stress indirectly affects medicinal plant growth and distribution by altering soil physicochemical properties and microbial community structure. Experiments with potted camphor trees revealed that severe drought lowered soil pH by 0.8 units and reduced organic matter content by 12.3%, while decreasing the relative abundance of soil actinomycetes and fungi by 28% and 35%, respectively [20]. Such changes diminish nutrient absorption efficiency in medicinal plant roots—for instance, licorice roots absorbed 21% less nitrogen in such soils. Imbalances in soil microbial communities also disrupt symbiotic relationships with medicinal plants [21]. The symbiotic efficiency between *Gastrodia elata* and *Armillaria mellea* decreased by 40% when soil moisture fell below 15% [13], thereby inhibiting tuber enlargement and active compound accumulation in *Gastrodia*. Additionally, elevated CO_2_ concentrations mitigate the negative impacts of extreme water stress by enhancing soil microbial activity. For instance, in chrysanthemum cultivation soils under high CO_2_ conditions, nitrogen-fixing bacteria abundance increased by 19%, restoring soil available nitrogen content by 8% and providing essential nutrients for medicinal plant growth [22].

**Figure 2:**
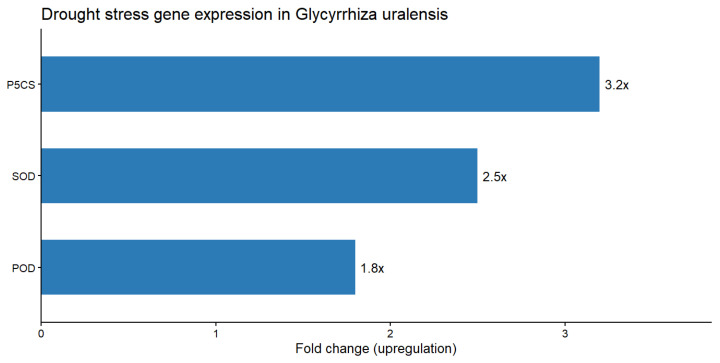
Upregulation of key stress-responsive genes in *Glycyrrhiza uralensis* under drought stress. The bar chart shows fold changes (relative to unstressed control) of three genes: *P5CS* (proline synthesis), *SOD* (superoxide dismutase), and *POD* (peroxidase). Data were measured in roots under moderate drought stress (soil moisture 40–45% field capacity). Higher fold change indicates stronger stress response.

At the molecular level, transcriptomics provides crucial insights into the genetic basis of stress resistance, revealing the molecular response mechanisms of medicinal plants to climate change. Under drought stress, licorice exhibited differential expression in 1248 genes. Among these, the *P5CS* gene associated with proline synthesis showed a 3.2-fold upregulation, while antioxidant system genes *SOD* and *POD* increased by 2.5-fold and 1.8-fold (as shown in Figure 2), respectively [23]. This enhanced drought tolerance through regulating osmotic adaptation and oxidative stress responses. Under diurnal temperature fluctuations, key volatile oil synthesis genes *DXS* and *HMGR* in patchouli exhibited rhythmic expression patterns. Nighttime low temperatures downregulated *DXS* expression by 40%, resulting in a 15% reduction in patchouli alcohol content [24]. Transcriptomic analysis further revealed that the *PgHSP01* gene in ginseng forms a regulatory network with the heat shock transcription factor *Hsfs*. Under high-temperature stress, they jointly regulate the saponin synthesis pathway, maintaining ginsenoside Rg1 content at over 70% of normal levels.

At a broader scale, frequent extreme climate events trigger restructuring of medicinal plant communities, thereby impacting regional herbal resource supply capacity. High-temperature summer droughts in the middle and lower Yangtze River regions reduced the community cover of authentic medicinal herbs like *Salvia miltiorrhiza* and *Scrophularia ningpoensis* by 30%, while increasing the invasion of extreme-climate-tolerant weeds like *Xanthium sibiricum* and *Achyranthes aspera* by 25%, diminishing the competitive advantage of medicinal plant populations [25]. The impact of elevated CO_2_ concentrations on medicinal plant communities exhibits interspecific variation. Among five chrysanthemum varieties under CO_2_ enrichment (800 μmol·mol^−1^), the biomass of “Golden Thread Imperial Chrysanthemum” increased by 22%, while that of “Hangzhou White Chrysanthemum” rose by only 7%. Such divergent growth responses alter the spatial distribution patterns of chrysanthemum varieties within communities [17]. Furthermore, extreme precipitation-induced flooding can cause soil salinization in authentic medicinal plant production areas. For instance, when soil salinity exceeds 0.3% in Hangbaiju cultivation zones, flavonoid content decreases by 28%, further accelerating the degradation of medicinal plant quality [26].

In summary, climate change exerts systemic impacts on medicinal plants by disrupting soil–microbe interactions, activating molecular regulatory networks, and reshaping communities. However, conflicting findings exist: moderate drought promotes flavonoid accumulation in *Scutellaria baicalensis* [27], but severe drought reduces it [21]; elevated CO_2_ boosts biomass in some chrysanthemum varieties but not in others [17]. Key knowledge gaps remain regarding multi-factor interactions, long-term responses, and field validation. Recent reviews emphasize the complex interplay between environmental factors and medicinal plant quality [7,28]. Existing studies have strengths in multi-omics applications [23,29] but limitations in short durations and single-factor designs. Addressing these gaps requires factorial experiments, long-term monitoring, and integration of molecular and ecological data. Understanding these responses is essential for conservation and adaptive management.

### 2.4. Indirect Impacts on Traditional Medicine Industries

Climate change impacts medicinal plants beyond the ecological realm, extending into socioeconomic and public health domains. It profoundly disrupts access to traditional medicines and modern pharmaceutical supply chains, triggering a series of cascading effects.

By compressing the suitable ranges of wild medicinal plants and reducing resource reserves and quality, climate change directly exacerbates the crisis of traditional medicine accessibility, with particularly significant impacts on ethnic minority communities dependent on indigenous medicinal plants. Among China’s ethnic medicinal plants, 28 endemic species in hotspots like Northwest Yunnan and Western Sichuan have experienced habitat shrinkage exceeding 50%. Essential wild ingredients for ethnic formulas—such as wild licorice and *Gastrodia*—have become significantly harder to collect, reducing the clinical application frequency of traditional therapies by 40% [30]. Climate warming and increased precipitation also alter nutrient accumulation in medicinal plants. Under conditions of 2 °C warming and 30% annual precipitation increase, the Mongolian medicinal herb Agui showed calcium and potassium content decreases of 18% and 12% respectively, while iron content rose by 9%. This significantly diminished therapeutic efficacy at traditional dosages, forcing some communities to abandon traditional treatment regimens. Within the Ailao Mountain National Nature Reserve, 32 nationally protected wild medicinal plant species have seen their core distribution altitudes rise by 300–500 m due to warming, with populations declining by over 40% [31]. Resource depletion in traditional collection areas has become acute. For economically underdeveloped regions and remote mountainous areas, the dual pressures of raw material shortages and price increases have created a dilemma where “prescriptions exist but medicinal materials are unavailable,” severely threatening the inheritance of traditional medicine and public health safeguards.

Simultaneously, climate change-induced fluctuations in medicinal plant yields, quality heterogeneity, and shifting production zones are causing systemic disruptions across the entire traditional Chinese medicine supply chain. In cultivation, extreme weather events have normalized reduced yields. For instance, droughts in Southwest China caused 25–35% declines in ginseng and cardamom production, while floods in the Yangtze–Huai River region led to over 40% drops in alisma production, inflicting severe economic losses on growers [25]. Regarding quality, temperature fluctuations and abnormal precipitation alter the secondary metabolic processes of medicinal plants. For instance, warming treatments reduced magnesium and calcium levels in the reproductive branches of Mongolian medicine *Agripaurus* by 3.6% and 3.9% respectively, while rain enhancement caused iron content to drop by 53.0% [26]. This poses challenges for raw material quality control in processed herbal slices and TCM formulation production. The distribution and sales channels have been impacted even more significantly. Unstable medicinal herb production has disrupted market supply and demand. For example, prices for herbs like *Platycodon grandiflorus* and *Codonopsis pilosula* affected by extreme weather surged in the Bozhou herbal market. Empirical data shows that for every one standard deviation increase in abnormal temperature fluctuations, the comprehensive price index for traditional Chinese medicinal materials rises significantly, with rhizome and flower herbs being the most sensitive to climate fluctuations [32]. Furthermore, the migration of authentic production areas has compelled some processing enterprises to relocate their production capacity. For instance, after the authentic production area of *Alisma orientale* shifted southward from the Yellow River basin, related enterprises incurred additional costs for equipment relocation and technical adjustments. Meanwhile, small and medium-sized merchants, unable to bear the risks of raw material price fluctuations, have exited the market, further exacerbating the vulnerability of the industrial chain [33].

In summary, climate change poses dual challenges to health service systems and economic systems based on medicinal plants by eroding traditional medicinal resource bases and disrupting modern industrial chains.

Based on the synthesized evidence, we summarize predicted changes for key species in Table 1.

## 3. Core Mechanisms of Climate Change Impacts on Medicinal Plants

### 3.1. Physiological and Biochemical Mechanisms

At the physiological and biochemical level, climate change profoundly impacts medicinal plants through metabolic regulation and stress responses. Core mechanisms include alterations in metabolic pathways governing active compound synthesis and heightened physiological damage due to oxidative stress system imbalances.

Climate change directly influences the accumulation of active compounds in medicinal plants by regulating the activity of key secondary metabolism enzymes. Flavonoid synthesis relies on phenylalanine ammonia-lyase (*PAL*), cinnamic acid 4-hydroxylase (*C4H*), and chalcone synthase (CHS), enzymes highly sensitive to climatic factors like temperature and light. During *Scutellaria baicalensis* seed germination, *PAL* and *C4H* activity peaked at 20 °C, with flavonoid content (e.g., baicalin, baicalein) significantly higher than other temperature gradients. High temperatures (35 °C) or low temperatures (15 °C) reduced enzyme activity by over 30% and total flavonoids by 60%. In mature *Scutellaria baicalensis* plants, enzyme activities of all three classes remained elevated at the optimal temperature range of 20–25 °C, with total flavonoid content 40–50% higher than in extreme temperature treatments [27].

Light regulates metabolism by promoting photosynthetic pigment synthesis. Increased light intensity and duration elevated *PAL* and *C4H* activities in *Scutellaria* seedlings by 30% and 25%, respectively, with concurrent increases in baicalin and wogonoside content. Low light, however, inhibited enzyme activity and secondary metabolite accumulation. Water stress alters primary biomass supply. Under drought, chlorophyll content decreased in *Scutellaria baicalensis* leaves, while baicalin content in roots increased with intensifying drought. Conversely, baicalin levels in stems and leaves plummeted under severe stress. Jujube leaf mesophyll cell photosynthetic activity is suppressed, with net photosynthetic rate (Pn) reduced by 45% compared to controls [36]. Insufficient carbohydrate synthesis indirectly limits secondary metabolism. Additionally, N, P, and K deficiencies caused 20–30% reductions in *PAL* and *C4H* activity in *Scutellaria baicalensis* roots. Appropriate potassium fertilization increased key enzyme activities by 15–20%, mitigating the inhibitory effects of climatic stress.

Concurrently, extreme climates trigger reactive oxygen species (ROS) bursts, disrupting antioxidant system equilibrium and causing lipid peroxidation damage to cell membranes. Under normal conditions, plants maintain cellular homeostasis by eliminating excess ROS via superoxide dismutase (*SOD*), catalase (CAT), and other enzymes. Under severe drought stress, *Scutellaria baicalensis* exhibited a 162% increase in leaf malondialdehyde (MDA) content compared to controls. with *SOD* and CAT activities initially rising then declining, failing to fully counteract ROS damage and compromising membrane integrity. Under severe water stress, *Ziziphus jujuba* leaves exhibited a 30% decrease in peroxidase (*POD*) activity compared to mild stress, an 89% increase in MDA content, and accelerated membrane lipid peroxidation. Temperature stress similarly disrupted stress responses. *Scutellaria baicalensis* seeds treated at 15 °C or 35 °C exhibited 20–30% higher *SOD*, *POD*, and CAT activities compared to the 25 °C control group. However, key secondary metabolism enzymes like *PAL* and *C4H* activity decreased, creating resource competition between stress protection and active compound synthesis. Under extreme temperatures, mature *Scutellaria baicalensis* accumulated large amounts of osmotic regulators like proline and soluble sugars. While this mitigated cellular dehydration, it displaced resources for secondary metabolite precursors, leading to reduced flavonoid content. Under drought stress, reduced stomatal conductance (Gs) and insufficient intercellular CO_2_ concentration (Ci) simultaneously inhibit the dark reaction of photosynthesis and exacerbate ROS accumulation through carbon metabolism imbalance, creating a vicious cycle of “photosynthetic inhibition–ROS burst–metabolic disruption [37]”.

In summary, climate change simultaneously disrupts the activity and coordination of key secondary metabolic enzymes while stimulating and potentially overwhelming plant antioxidant defense systems. This dual interference shapes the physiological and biochemical response patterns of medicinal plants at both synthetic and degradative levels, ultimately determining their quality and stress tolerance. Notably, the relationship between stress intensity and metabolite accumulation is often non-linear, with moderate stress promoting bioactive compounds while severe stress suppresses them, and species vary widely in their temperature thresholds for enzyme inactivation.

### 3.2. Molecular Genetic Mechanisms

Climate change impacts medicinal plants at the molecular level, shaping their genetic response patterns to environmental stress through dual regulation of gene expression and epigenetic modifications.

Climate change induces enrichment of differentially expressed genes in medicinal plants, regulating secondary metabolism and stress-related pathways, thereby influencing active compound synthesis and stress adaptation. Under drought stress, transcriptomic analysis of *Asarum sieboldii* identified 6444 differentially expressed genes among 53,344 assembled single genes, primarily enriched in phenylpropanoid, starch, and sucrose metabolism pathways. Key genes such as *PAL*, *C4H*, and *HCT* in the methyl eugenol synthesis pathway showed significant upregulation. Prolonged drought reduced the total volatile oil content in *Asarum*, yet paradoxically increased the yield of the core bioactive compound methyl eugenol, demonstrating selective gene expression regulation of active compounds [29]. MYB transcription factor family members in *Rheum palmatum* participate in stress response and metabolic regulation. *RhMYB1*, *RhMYB2* showed significant upregulation under drought and high-temperature stress. Notably, *RhMYB3* and *RhMYB4* interacted with genes involved in the synthesis of anthraquinones such as emodin and aloe-emodin [34], mitigating stress-induced suppression of bioactive compound accumulation by activating downstream metabolic pathways. Similarly, a study on *Panax ginseng* showed that the MeJA-responsive transcription factor *PgMYB2* positively regulates dammarenediol synthase, a key enzyme in ginsenoside biosynthesis [38]. Additionally, under high-temperature stress, the expression of heat shock protein genes in *Pinellia ternata* significantly increased, while key alkaloid synthesis genes were suppressed, leading to reduced levels of bioactive compounds such as guanosine and hypoxanthine [39].

DNA methylation, as a core epigenetic modification, mediates the adaptive response of medicinal plants to climatic stress by regulating gene expression patterns. Under high-temperature stress, the genomic DNA methylation level in *Pinellia* significantly increased, with a marked rise in methylation rates at CG sites compared to the control. Methylation modifications directly influence the expression of stress-resistant and metabolic genes. Under high-temperature stress, the expression levels of antioxidant enzyme genes such as *SOD* and *POD* in *Pinellia ternata* decreased due to elevated methylation levels [39]. Different climatic factors exert specific effects on epigenetic modifications. Under drought stress, the genomic DNA methylation patterns of *Rheum palmatum* undergo significant alterations, with differentially methylated sites in promoter regions predominantly enriched in stress-related genes and genes involved in anthraquinone compound synthesis [40]. This methylation modification exhibits a degree of reversibility. Upon stress relief, methylation levels of some genes return to normal, and their expression levels are consequently re-regulated, enabling medicinal plants to maintain metabolic homeostasis while adapting to environmental changes [41]. Furthermore, epigenetic modifications such as histone acetylation and phosphorylation synergize with DNA methylation. By regulating chromatin structure, they achieve fine-tuned control over stress-response and metabolic genes, enhancing medicinal plants’ adaptability to climate change [42].

Beyond DNA methylation, emerging evidence indicates that microRNAs (miRNAs) and histone modifications play critical roles. For example, drought-induced *miR393* targets auxin receptor genes to regulate root architecture in medicinal plants, while histone H3 acetylation at the promoter regions of heat shock protein genes facilitates rapid transcriptional activation under heat stress. These additional epigenetic layers should be prioritized in future research. Across studies, a convergent observation is that diverse abiotic stresses converge on a limited set of transcription factor families (MYB, WRKY, NAC), suggesting a conserved “stress-regulatory module” in medicinal plants.

### 3.3. Ecological Interaction Mechanisms

Climate change not only directly impacts individual medicinal plants but also triggers cascading effects at the community and ecosystem levels by disrupting their complex interaction networks with biotic and abiotic environments.

Climate change has caused temporal and spatial mismatches between the activity periods of pollinating insects and the flowering seasons of medicinal plants, directly impacting pollination efficiency and population reproduction. The peak flowering period of the Ningqi No. 10 goji berry variety originally aligned closely with the peak foraging period of primary pollinators like honeybees. However, rising temperatures have caused the goji berry flowering period to advance, while the onset of bee activity has not synchronized accordingly, leading to reduced fruit set rates [43]. Beyond pollinator mismatches, climate change also facilitates pathogen expansion and invasive plant competition. Under high-temperature and high-humidity conditions, the spore germination rate of rice blast pathogens increases, shortening the incubation period and significantly raising disease incidence in medicinal rice varieties [44]. Warmer winters allow pine bark beetle populations to survive at higher elevations, expanding their range into previously unexposed medicinal plant habitats. Simultaneously, extreme climate events (e.g., summer drought in the Yangtze region) reduce the competitive advantage of native medicinal plants such as *Salvia miltiorrhiza*, while promoting the invasion of climate-tolerant weeds like *Xanthium sibiricum* and *Achyranthes aspera*, which increased by 25% in invaded plots. Drought stress further reduces plant resistance, making them more susceptible to pest attacks. Since the reproduction rate of natural enemies lags behind that of pests, the severity of damage intensifies [45].

Climate change significantly reshapes soil microbial community structures, disrupting symbiotic relationships between medicinal plants and beneficial microorganisms and reducing nutrient absorption efficiency. Rising temperatures reduce bacterial community diversity in soil while increasing the proportion of pathogenic fungal groups, diminishing the symbiotic efficiency between mycorrhizal fungi and medicinal plants [46]. Under drought stress, the infection rate of arbuscular mycorrhizal fungi in amaranth rhizosphere decreases, reducing plant efficiency in absorbing nutrients like phosphorus and nitrogen [47]. Altered soil microbial functions also indirectly impact medicinal plant growth. Under drought conditions, reduced soil urease and sucrase activity slows organic matter decomposition, diminishing available carbon and nitrogen sources for plant roots [48]. Extreme weather events further destabilize habitat microenvironments. Heavy rainfall causes soil compaction, reducing root aeration and lowering milkweed seed germination rates. Persistent drought reduces soil moisture content, disrupting the symbiotic nitrogen-fixing system between roots and rhizobia, resulting in fewer nodules on *Astragalus membranaceus* roots and decreased biomass accumulation [49]. Furthermore, changes in soil pH affect microbial metabolic activity, significantly reducing the abundance of beneficial microbial communities in the rhizosphere of medicinal plants, thereby further exacerbating nutrient limitations.

## 4. Advances in Research Methods for Medicinal Plant Responses to Climate Change

### 4.1. Observational and Experimental Methods

Modern research methods integrate field monitoring, controlled experiments, and isotope tracing techniques to establish a multi-scale analytical system spanning macro-ecological phenomena to micro-physiological mechanisms.

Field monitoring has evolved from traditional manual phenological recording to an IoT-driven, precision-based, real-time monitoring system, enabling dynamic tracking of medicinal plant growth environments and physiological states. Environmental control systems based on LoRa wireless communication and PLC control integrate multiple sensors—including soil NPK, temperature/humidity, CO_2_ concentration, and light intensity—via RS-485 bus. These systems enable wireless data transmission within 3 km at ≤10 s sampling intervals. Combined with MQTT protocol, this data is pushed to cloud platforms, providing high-frequency support for analyzing habitat dynamics of medicinal plants under climate change [50]. In long-term fixed-point observations, standardized plots are established to continuously track phenological stages (e.g., flowering and harvest timing)growth indicators (plant height, biomass), and environmental parameters. For instance, continuous monitoring of *Pinellia ternata*’s growth under high-temperature stress reveals the dynamic relationship between DNA methylation levels and lodging rates [39]. Additionally, IoT terminals in smart greenhouses (e.g., outdoor weather stations, indoor environmental sensors) enable long-term monitoring of physiological parameters like photosynthetic rates and transpiration rates in medicinal plants, providing foundational data for quantifying climate change impacts on plant growth [51].

Controlled experimental methods become central to deciphering the multifactorial interactions of climate change. Controlled climate chamber experiments emerge as a core approach for analyzing these interactions, enabling precise regulation of key factors like temperature, precipitation, and CO_2_ concentration to quantify the mechanisms by which single or combined stresses affect medicinal plants. For instance, by setting different temperature gradients (25 °C, 30 °C, 35 °C) and CO_2_ concentration levels (400 ppm, 800 ppm), future climate scenarios were simulated to investigate their effects on the activity of key enzymes involved in the synthesis of flavonoids in *Scutellaria baicalensis* (*PAL*, *C4H*) [27]. In multifactorial interaction studies, combining drought (PEG simulation), salinity (NaCl gradient), and acid stress (pH gradient) treatments clarified the response patterns of milkweed and amaranth seed germination rates, vitality, and recovery characteristics to combined stress [47]. Isotope labeling techniques provide direct evidence for tracing the synthesis pathways of bioactive compounds. For instance, ^13^C labeling technology tracks the conversion of photosynthetic products into bioactive components of medicinal plants (e.g., Lycium polysaccharides, emodin), revealing the linkage between carbon metabolism shifts under climate change and the accumulation of bioactive compounds [48].

### 4.2. Modeling and Prediction Methods

Modeling approaches achieve quantitative simulation and future projections of medicinal plant responses to climate change by integrating physiological processes, environmental factors, and spatial distribution.

Physiology–climate coupling models integrate plant physiological metabolism with climate drivers. For instance, the APSIM model has been parameterized for ginseng yield simulation, quantifying yield responses under warming scenarios. A dedicated model developed for medicinal yam simulates the northward expansion of suitable zones under future climate conditions. Such models enhance the mechanistic accuracy of active compound accumulation simulations by incorporating photosynthesis and secondary metabolism modules [52].

Ecological suitability models focus on analyzing species distribution patterns in relation to environmental factors. MaxEnt models, combined with Geographic Information System (GIS) technology, integrate distribution point data with climate and soil factors to delineate current suitable areas and identify dominant environmental drivers. Model applications have expanded to future scenario projections, where coupling with climate model outputs reveals how climate change reshapes suitable area extent and spatial patterns. To ensure reproducibility, future scenario projections must explicitly report the climate model source, scenario, time slice, and spatial resolution.

Model calibration and validation emphasize multi-source data integration and accuracy control. Parameter calibration relies on field measurements, using optimization algorithms to derive key physiological parameters. Independent validation requires strict error criteria to ensure simulation reliability. The data support system encompasses climate scenario data, field physiological indicators, and soil attribute databases. Some studies promote methodological standardization by releasing models and data publicly.

### 4.3. Omics Technologies and Methods

The rapid advancement of omics technologies provides powerful tools for systematically deciphering the molecular mechanisms underlying medicinal plant responses to climate change, spanning multiple dimensions including genomics and metabolomics.

Genomics technologies provide core support for genetic analysis and resource development of medicinal plants. By integrating third-generation long-read and second-generation short-read sequencing technologies, supplemented with chromosome conformation capture techniques like Hi-C, high-quality genome assembly at the chromosomal level can be achieved [53]. Building upon this foundation, comparative genomics analysis can elucidate phylogenetic relationships, evolutionary histories, and events such as whole-genome duplications across species. It also identifies key gene families involved in secondary metabolite synthesis and stress responses, along with their expansion and contraction patterns. Functional genomics further unearths key enzyme genes and regulatory factors participating in specific bioactive compound biosynthetic pathways through gene annotation and expression analysis.

Metabolomics technologies enable precise, high-throughput analysis of metabolite profile shifts in medicinal plants under climate change stress. Non-targeted metabolomics strategies, combined with gas chromatography–mass spectrometry (GC-MS) or liquid chromatography–high-resolution mass spectrometry (LC-HRMS) platforms, systematically identify and relatively quantify numerous small-molecule metabolites in biological samples. Multivariate statistical methods such as princi*PAL* component analysis and orthogonal partial least squares discriminant analysis effectively distinguish samples from different treatment groups or sources and identify significantly altered differential metabolites [54]. Further metabolic pathway enrichment analysis helps reveal potential biological processes and metabolic network perturbations influenced by climate change. Recent multi-omics studies have demonstrated the power of these approaches: for example, integrated transcriptomic and metabolomic analysis in *Astragalus membranaceus* under cold stress identified WRKY transcription factors as key regulators of flavonoid biosynthesis [55], while combined genomic and transcriptomic profiling in *Rheum palmatum* uncovered a drought-activated anthraquinone biosynthetic gene cluster [53]. These findings illustrate how omics technologies can pinpoint molecular targets for climate adaptation breeding.

### 4.4. Methodological Comparisons and Limitations

In studies examining medicinal plant responses to climate change, all methodologies require scientific evaluation, acknowledging their inherent strengths and limitations.

While MaxEnt modeling is widely used for habitat suitability prediction, it often lacks microclimate, soil property, and human activity data, leading to potential overestimation or underestimation of actual refugia. Omics technologies (genomics, transcriptomics, metabolomics) provide powerful molecular insights, but multi-omics integration remains a bottleneck due to high data dimensionality, poor comparability across studies, and insufficient field validation. Controlled environment chambers allow precise manipulation of single factors but may not reproduce the complexity of field conditions where multiple stresses co-occur.

Quantitative assessment tools are essential for evaluating resource utilization sustainability. For instance, the Pattern–Pathway Model establishes an analytical framework linking “resource utilization patterns” with “evolutionary pathways,” enabling systematic evaluation of medicinal plant resource supply system stability, sensitivity to external changes, and future sustainability. This provides quantitative evidence for formulating resource management strategies.

Future methodological advances should prioritize: (i) coupling models with high-resolution microclimate data; (ii) establishing open-access benchmark datasets for multi-omics integration; and (iii) designing field-based factorial experiments that mimic realistic combined stress scenarios.

## 5. Case Studies of Representative Regions and Species

### 5.1. Regional Case: Asia vs. Europe, America, and Africa Comparison

Research on medicinal plants under climate change exhibits distinct regional characteristics globally, with Asia and Europe/America/Africa showing marked differences in research focus, depth, and application directions.

Asian research centers on habitat shifts affecting authentic medicinal materials, quality formation regulation, and stress resistance mechanisms in traditional species, demonstrating strong systematic and application-oriented approaches. As a core research area, China focuses on renowned authentic medicinal materials like rhubarb and ginseng, comprehensively employing modern technologies such as genomics and metabolomics to deeply analyze the biosynthetic pathways of their active components and their intrinsic links to environmental adaptability. For instance, research on Lijiang rhubarb (*Rheum palmatum*) through chromosome-level genome assembly identified key gene families involved in anthraquinone synthesis and their expression patterns. Systematic exploration of the chemical constituents and biological activities of the endemic species *Abies fargesii* laid the foundation for new drug development [56]. India focuses on the response mechanisms of traditional medicinal plants under drought stress. Through physiological-ecological and molecular-level experiments, it reveals their metabolic regulation strategies, providing theoretical support for resource conservation in arid regions.

In contrast, European research exhibits a distinct “distribution-oriented” approach, concentrating on the geographic distribution dynamics of alpine medicinal plants (e.g., Valeriana) under climate change. Studies primarily rely on long-term field monitoring and model simulations to analyze how factors like temperature and precipitation influence species ranges, though investigations into deeper mechanisms—such as changes in active compounds and molecular adaptation pathways—remain relatively limited. Research in Africa and South America lags behind due to constraints imposed by insufficient baseline data. Related studies predominantly concentrate on a limited number of economically valuable species, primarily involving resource baseline surveys and documentation of traditional application knowledge. There is a widespread lack of systematic chemical component isolation and identification, validation of pharmacological activity, and analysis of environmental adaptation mechanisms. In terms of research depth and translational application, there is a significant gap compared to the Asian region [57]. These disparities stem from four interacting drivers: climate regimes (monsoon vs. Mediterranean vs. semi-arid), conservation policies (China has Dao-di herb protection; Africa lacks medicinal plant legislation), cultivation systems (intensive Asian farming vs. European wild collection), and research infrastructure (high-throughput omics are concentrated in Asia and parts of Europe). Addressing these imbalances requires international collaboration and capacity building.

### 5.2. Species Case Study: Climate-Sensitive Medicinal Plants

Medicinal plants across different ecological types exhibit species-specific responses to climate change, with high-altitude species, authentic medicinal materials, and endangered species facing distinct challenges and adaptation strategies. To provide a more structured comparison, we categorize climate-sensitive medicinal plants into three priority types: (1) high-altitude alpine species (e.g., *Nardostachys jatamansi*, *Rhodiola rosea*) [58], which are constrained by narrow thermal niches and show upward migration; (2) geo-authentic herbs (e.g., *Rheum palmatum*, *Panax ginseng*), whose quality is tightly linked to specific soil–climate combinations; and (3) endangered species (e.g., *Paris polyphylla*, *Cordyceps sinensis*), facing compounded threats from climate stress and overharvesting. The following subsections provide representative examples from each category.

High-altitude medicinal plants exhibit heightened sensitivity to climate change. Represented by *Himalayan costus*, its growth relies on alpine shrublands and grasslands at 2600–5000 m elevation. Climate change has caused 30% habitat loss, resulting in a significant 22% decline in core active compound content [58]. As a distinctive Tibetan medicinal herb, its primary active components are sesquiterpenes (e.g., calamone) and essential oils, whose synthesis and accumulation are closely linked to high-altitude conditions such as low temperatures and intense sunlight. Rising temperatures not only compress its suitable altitude range but also disrupt the synthetic balance of volatile oils and terpenoids, weakening pharmacological activities like sedation and antiarrhythmia. Simultaneously, excessive harvesting exacerbates resource depletion, leading to its listing in the IUCN Red List of Threatened Species [59].

Significant climatic adaptation differentiation exists among authentic medicinal plant populations. Studies on the *Rheum palmatum* complex reveal climate change-driven genetic structural variation. Through whole-genome resequencing and genotype–environment association analysis, two subspecies lineages were identified: western and eastern. The western lineage, having long adapted to variable climates, possesses 16,709 pan-adaptive sites and 1198 core adaptive sites, indicating stronger pre-adaptive potential [35]. The eastern lineage exhibits high genomic drift and lower genetic diversity, facing greater maladaptation risks and requiring enhanced climate resilience through “auxiliary gene flow” strategies. As a classic authentic medicinal herb, Changbai Mountain ginseng’s quality formation is closely linked to meteorological factors. The grey correlation coefficients between average temperature, precipitation, and sunshine duration during the growing season and total saponin content range from 0.756 to 0.781. Total saponins peak at 26.64 mg/g during optimal climatic conditions from late September to early October, marking the optimal harvest period [60].

Endangered medicinal plants face dual threats from climate stress and human disturbance. Wild *Paris polyphylla* populations have declined by 45% due to extreme precipitation, habitat fragmentation, and predatory harvesting. Paris thrives in shaded, moist understories of subtropical montane broadleaf forests at 500–1000 m elevation, exhibiting high dependence on cool, humid habitats rich in humus. Changes in precipitation patterns and temperature fluctuations caused by climate change, combined with its biological characteristics of seed “secondary dormancy” and a long reproductive cycle (over 5 years), have accelerated population decline [61]. Although artificial cultivation has gradually been implemented, it has yet to overcome the bottleneck of climate adaptability. *Paris polyphylla* cultivated in low-altitude areas suffers from insufficient saponin content and poor growth and development, making it difficult to replace the ecological and medicinal value of wild populations. Beyond these categories, medicinal plants from other habitats also face distinct threats: wetland species (e.g., *Alisma orientale*) are highly sensitive to precipitation changes [33], while desert-adapted species (e.g., *Glycyrrhiza uralensis* in arid regions) show tolerance to moderate drought but suffer under extreme heat [21]. Expanding research to cover halophytes, psammophytes, and aquatic medicinal plants would provide a more comprehensive understanding of climate adaptation across diverse ecological niches.

## 6. Adaptive Management Strategies and Research Outlook

### 6.1. Core Adaptation Strategies

In ecological cultivation models and soil microecological regulation, intercropping and relay cropping not only alleviate continuous cropping obstacles for medicinal herbs but also enhance the biodiversity and stability of agricultural systems, thereby improving medicinal plants’ adaptability to climate change. For instance, *Platycodon grandiflorus*–*Allium fistulosum* intercropping optimizes soil microbial structure, increases beneficial microbial communities, suppresses pathogenic fungi, and helps maintain soil health, enhancing crop disease resistance and resilience under climate fluctuations. The *Angelica sinensis*–garlic intercropping system significantly elevates soil enzyme activity, enhances nutrient cycling efficiency, and supports crop physiological metabolism under drought or high-temperature stress. The *Pinellia ternata*–soybean intercropping system promotes soil nutrient accumulation [62], increases yields, and provides a buffer against yield fluctuations caused by climate change.

Precision water and fertilizer management systems directly address climate-induced water imbalance and nutrient loss by precisely regulating moisture and nutrient supply. For instance, leaf-use wolfberries demonstrated significantly improved water and nutrient use efficiency under optimized water–fertilizer ratios, enhancing growth stability and secondary metabolite accumulation under drought or high-temperature conditions [63]. Precision management tailored to the varying water requirements of different medicinal plants can effectively mitigate abiotic stresses exacerbated by climate change, such as drought and waterlogging.

In terms of stress-resistant gene discovery and mechanism analysis, technologies like transcriptomics identify stress-resistant genes, providing a molecular foundation for breeding medicinal plant varieties adapted to climate change. Defense protein and lipoprotein genes identified in ginseng not only enhance disease resistance but also improve tolerance to multiple climatic stresses like salinity and drought [64]. These genes establish a defense network against combined climatic stresses by regulating antioxidant systems and osmoregulatory substance synthesis, thereby boosting the survival and yield stability of medicinal plants in changing environments. Emerging strategies—assisted migration, conservation genetics, and climate-smart cultivation—should also be integrated into adaptive management.

### 6.2. Future Research Prospects

Four priority directions for future research are: (1) long-term field experiments, (2) multi-factor climate stress studies, (3) integration of omics with ecological observations, and (4) validation of predictive models under real-world conditions. As global climate change intensifies, medicinal plant resources face unprecedented challenges [2]. Future research will focus on three dimensions: unraveling climate stress mechanisms, integrating multi-omics technologies, and fostering interdisciplinary collaboration. This will establish a systematic research paradigm of “mechanism exploration–technological innovation–application integration.” This approach goes beyond observing the surface effects of climate change on medicinal plant growth, delving into multi-level response mechanisms from gene regulation to ecosystems. Technological innovations will drive the translation of scientific discoveries into practical applications.

Regarding key scientific questions, research must elucidate how extreme climate events and soil environmental changes synergistically influence secondary metabolic pathways in medicinal plants, revealing the patterns by which these complex interactions shape medicinal yield and quality. At the molecular level, there is an urgent need to construct dynamic association models linking gene expression regulation, metabolic network reorganization, and physiological adaptation responses [23]. This will clarify the mechanisms by which key regulatory elements, such as WRKY transcription factors, contribute to stress resistance in medicinal plants [55]. Concurrently, the regulatory logic of ecological cultivation and precision management must be systematically elucidated from the perspective of soil–plant–microbe interactions, providing theoretical guidance for environmental adaptation in medicinal production.

Technological innovation serves as the core driver for multidisciplinary synergy. The refinement of integrated transcriptomics and metabolomics technologies promises to enable detailed analysis of secondary metabolic pathways across a broader range of medicinal plants. The application of cutting-edge techniques like single-cell sequencing will enhance the spatiotemporal resolution of stress-resistant gene discovery, providing precise targets for molecular breeding. Advancements in intelligent monitoring and multi-source data fusion technologies enable optimization of technical solutions such as precision water and fertilizer management and green control of continuous cropping obstacles. Improvements to models like CA–Markov provide more reliable quantitative tools for medicinal plant cultivation zoning [65].

Multidisciplinary integration opens new avenues for the sustainable development of medicinal plant resources. The deep integration of biology and agronomy drives innovation across the entire chain from genes to field management, while the intersection of ecology and soil science provides a theoretical foundation for soil health and efficient nutrient utilization. The convergence of information science and agronomy has spawned intelligent cultivation management technologies [66], while the synergy between pharmacology and environmental science has established a comprehensive evaluation system spanning “ecological environment–cultivation process–herbal quality”.

## 7. Conclusions

### 7.1. Summary of Evidence

This scoping review of 65 articles yields three core scientific insights: (1) Climate change drives consistent range shifts and phenological disruptions across medicinal plant species, with bioactive component fluctuations ranging from –50% to +20% in a species- and stress-specific manner. (2) The underlying mechanisms operate at three integrated levels—physiological (ROS imbalance, enzyme activity changes), molecular (differential gene expression, DNA methylation, transcription factor networks), and ecological (pollinator mismatches, soil microbiome shifts). (3) Existing conservation efforts are skewed toward ex situ protection, with a critical gap in climate-smart adaptive management and assisted migration strategies.

Practically, these findings underscore the need to incorporate medicinal plant conservation into climate adaptation agendas, develop climate-resilient cultivation systems, and prioritize multi-factor and long-term field studies. The proposed “Environment–Gene–Quality” framework offers a testable model for future research across different socio-ecological contexts.

### 7.2. Limitations

This scoping review has several limitations. First, the literature search was limited to articles published in English and Chinese, which may have introduced language bias. Second, no formal critical appraisal of methodological quality was performed, consistent with the scoping review objective of mapping the breadth of evidence; however, this means that the quality and validity of individual studies were not assessed. Third, the search was restricted to publications from 1994 onwards, and duplicate records were not removed, which may have affected the comprehensiveness of the evidence synthesis. Fourth, the geographical distribution of included studies was heavily skewed toward Asia (particularly China), with fewer studies from Europe, North America, and Africa; therefore, the findings may not be fully generalizable to other regions. Finally, as with any scoping review, the results are current only up to the search date (May 2026).

### 7.3. Implications and Future Research

The findings of this review have several implications. For policymakers, the results highlight the urgent need to incorporate medicinal plant conservation into climate adaptation agendas, particularly for high-altitude and endemic species. For researchers, the proposed “Environment–Gene–Quality” framework offers a testable model for future studies. For the pharmaceutical industry, the documented 20–50% fluctuations in bioactive components underscore the need for climate-resilient cultivation and quality control systems.

Future research should prioritize: (1) compound stress experiments to unravel non-additive effects of combined climate factors; (2) multi-omics integration coupled with field validation to bridge the gap between molecular mechanisms and real-world responses; (3) development and testing of the “Environment–Gene–Quality” framework across different socio-ecological contexts; and (4) long-term monitoring studies to track genetic and functional diversity dynamics under changing climates.

## Figures and Tables

**Table 1:** Predicted changes in bioactive compounds of representative medicinal plants under 1.5 °C global warming scenario (based on experimental and modeling studies).

Species	Bioactive Compound(s)	Predicted Change Under +1.5 °C
*Scutellaria baicalensis*	Baicalin, wogonoside	Increase at 20–25 °C (optimal), decrease > 35 °C [27]
*Glycyrrhiza uralensis*	Glycyrrhizic acid, liquiritin	Increase under moderate drought; decrease under severe heat [21]
*Panax ginseng*	Ginsenoside Rg1	Maintain > 70% of normal via heat shock protein regulation [19]
*Gastrodia elata*	Gastrodin	Decrease (>32%) under combined heat and drought [13]
*Rheum palmatum*	Emodin, aloe-emodin	Complex, mediated by MYB transcription factors [34,35]

Note: Predicted changes are based on experimental data from the cited references, assuming a +1.5 °C temperature rise above pre-industrial levels, often combined with associated changes in precipitation or CO_2_.

## Data Availability

No new data were generated for this review. All data discussed are from publicly available sources cited in the reference list.
